# Volatile Oil of* Amomum villosum* Inhibits Nonalcoholic Fatty Liver Disease via the Gut-Liver Axis

**DOI:** 10.1155/2018/3589874

**Published:** 2018-07-19

**Authors:** Shanhong Lu, Ting Zhang, Wen Gu, Xingxin Yang, Jianmei Lu, Ronghua Zhao, Jie Yu

**Affiliations:** College of Pharmaceutical Science, Yunnan University of Traditional Chinese Medicine, Kunming 650500, China

## Abstract

**Background:**

The dried mature fruit of* Amomum villosum *has been historically used in China as food and in the auxiliary treatment of digestive system disorders. Numerous studies have shown that gastrointestinal function is closely related to the development of nonalcoholic fatty liver disease via the “gut-liver” axis.

**Objective:**

The present study aimed to explore whether the mechanism underlying the regulation of lipid accumulation in nonalcoholic fatty liver disease (NAFLD) may affect related disorders using the active ingredients in* A. villosum*.

**Design:**

Male Sprague-Dawley rats on a high-fat diet (HFD) to induce NAFLD were administered water extract of* A. villosum *(WEAV), volatile oil of* A. villosum *(VOAV), or bornyl acetate. After treatment, serum and liver total cholesterol (TC), triglyceride (TG), free fatty acid (FFA), aspartate aminotransferase (AST), alanine aminotransferase (ALT), high-density lipoprotein cholesterol (HDL-C), and low-density lipoprotein cholesterol (LDL-C) levels were measured. The regulatory role of* A. villosum *in the microecology of the intestines was assessed using the V4 region of the 16S rDNA sequencing. The expression of the intestinal tight junction proteins occludin and ZO-1 was also measured. The influence of* A. villosum* on TLR4-mediated chronic low-grade inflammation was evaluated based on the concentrations of key proteins of the TLR4/NF-кB signaling pathway.* Results. A. villosum* effectively inhibited endogenous lipid synthesis, reduced TG, TC, and FFA accumulation, regulated the expression of LDL-C, and decreased lipid accumulation in liver tissues. VOAV effectively regulated the intestinal microflora, improved chronic low-grade inflammation by promoting ZO-1 and occludin protein expressions, and inhibited the TLR4/NF-кB signaling pathway.

**Conclusion:**

The present study provides scientific basis for the potential application of* A. villosum* in NAFLD prevention and treatment. Additional chemical constituents other than bornyl acetate also contributed to the preventive effects of* A. villosum* on NAFLD.

## 1. Introduction

Nonalcoholic fatty liver disease (NAFLD) is a common disorder that is characterized by accumulation of excess fats in the liver of individuals who drink little or no alcohol. Epidemiological studies have shown that NAFLD may affect individuals of any age and race. The prevalence of NAFLD in Western countries is about 18%–35%, and up to 80% of obese individuals develop NAFLD [1]. NAFLD is a progressive disease that results in irreversible liver injury and may also be a risk factor for liver fibrosis and cancer [2]. Recent studies have focused on elucidating the role of the intestinal microbial environment and its feedback effects on the liver in the pathogenesis of NAFLD [3, 4]. Excess uptake of free fatty acids (FFA) from food may lead to disorders of the intestinal microbial system. The intestinal microbial environment affects fat deposits in liver cells, which may be involved in the early pathogenesis of NAFLD. The relative balance of intestinal microflora significantly affects the absorption of fatty acids and other nutrients, and thus changes in the intestinal microbial environment and feedback may influence the pathogenesis of liver diseases [4, 5]. The gut microbiota of obese humans and HFD-fed mice was characterized by higher Firmicutes-to-Bacteroidetes ratios, an increase in the number of endotoxin-producing Proteobacteria, and a reduction in the number of immuno-homeostatic bacterial species [6, 7]. Intestinal microbial balance disorders [8], small intestinal bacterial overgrowth (SIBO) [9], changes in intestinal permeability [10], serum lipopolysaccharide (LPS) overload (endotoxemia) [11], and subsequent events are closely related to each other and are considered indicators of NAFLD onset [12, 13] and collectively called the gut-liver axis. The efficacy and mechanism of action of treatment regimens for NAFLD are largely associated with the intestinal hepatic axis. However, no effective pharmacotherapeutic regimen for NAFLD has been established to date. Despite advances in establishing its natural history, the underlying mechanism and pathogenesis of NAFLD remain elusive [14]. Exercise and diet are the most basic therapeutic interventions for NAFLD; however, this has been determined to be insufficient [15]. Hence, a highly specific and effective drug treatment is warranted.

Amomi Fructus is the dry, mature fruit of* Amomum villosum* Lour.,* A. longiligulare* T.L. WU, and* A. villosum *Lour. var.* xanthioides* T.L. Wu et Senjen of Zingiberaceae [16]. Previous studies have indicated that Amomi Fructus has the capacity to regulate gastrointestinal flora [17]. Volatile oil of* A. villosum *significantly inhibited the growth of* Trichophyton rubrum*,* T. mentagrophytes*,* Microsporum gypseum*,* Staphylococcus aureus*, and* Enterococcus faecalis* [18]. Furthermore, research studies have shown that Amomi Fructus extracts can prevent alcohol-induced lipid oxidation in liver cells and activate alcohol dehydrogenase, aldehyde dehydrogenase, and* CYP2E1* gene expression [19, 20]. However, the mechanism of action of* A. villosum* in the treatment of NAFLD remains unclear.

Recent studies have focused on elucidating the relationship between intestinal flora and NAFLD. The present study is the first to focus on the role of different* A. villosum* extracts in the regulation of intestinal microflora, adjustment of intestinal microflora equilibrium, control of low-grade chronic inflammation, reduction of fat accumulation, and consequent alleviation of NAFLD, hyperlipidemia, and related lipid metabolism disorders. Our findings indicate that* A. villosum* could effectively inhibit endogenous lipid synthesis, reduction of TG, TC, and FFA accumulation, regulation expression of LDL-C, and reduction of lipid accumulation in liver tissue. VOAV could effectively regulate the intestinal microflora, relieve chronic low-grade inflammation by promoting ZO-1 and occludin protein expressions, and inhibit the TLR4/NF-кB signaling pathway. VOAV showed great potential in NAFLD prevention and treatment. Possible major active constituents and dose-effect relationships of* A. villosum* were also evaluated in this study to provide evidence for its efficacy in clinic applications.

## 2. Materials and Methods

### 2.1. Plant Materials and Chemicals

The fruits of* A. villosum* Lour. were collected in June 2014 from Jinping County, Honghe Prefecture, Yunnan Province, China, and identified by Professor Ronghua Zhao, Yunnan University of Traditional Chinese Medicine. Voucher specimens (Specimen number: LSH 20140605) were deposited in the Herbarium of the Traditional Chinese Medicine Pharmacognosy Department of Yunnan University. Bornyl acetate (BA, purity: > 98%), the main constituent in volatile oil of* A. villosum* Lour [4], was purchased from Nanjing Jingzhu Biotechnology Co., Ltd., China. Ezetimibe tablets (Hangzhou MSD Pharmaceutical Co., Ltd., Hangzhou, China) were used as positive control.

### 2.2. Preparation of Water Extract of* A. villosum* (WEAV) and Volatile Oil of* A. villosum* (VOAV)

Powder (100 g, 20 mesh) of* A. villosum* fruit was weighed and immersed in water for 30 min (4°C). It was then refluxed thrice with 500 mL of water for 30 min each time. These water extracts were pooled, concentrated, and lyophilized. The final extraction rate of WEAV was 23.56% of the crude drug.

VOAV was extracted from the powdered crude drug by steam distillation according to the procedure recorded in the Chinese Pharmacopoeia (2015 edition) [5]. The powdered (100 g, 20 mesh) fruit of* A. villosum* was weighed and soaked in water for 12 h (4°C), and then steam distillation was performed with 800 mL of water for 6 h. The final extraction rate of VOAV was 3.90% of crude drug.

### 2.3. Quality Control of WEAV as Indicated by High-Performance Liquid Chromatography with Diode-Array Detection (HPLC-DAD)

Flavonoids and organic acids are considered major nonvolatile constituents of* A. villosum *[21, 22]. Thus, HPLC determinations for quercitrin, isoquercitrin, and vanillic acid (4-hydroxy-3-methoxybenzoic acid) in WEAV were performed in this study.

All experiments were conducted using a Dionex Ultimate 3000 HPLC system (Dionex Technologies, Sunnyvale, California, USA). Data were analyzed with Chromeleon 6.8. These compounds were separated by a Nucleodur C18 Gravity column (4.6 mm × 250 mm, I.D., 5 *μ*m, Agilent Technologies, USA). The gradient elution used a mobile phase consisting of (A) 0.4% H_3_PO_4_ and (B) methanol. The following gradient program was used: 20% B (0 min), 30% B (25 min), 40% B (30 min), 50% B (40 min), and 50% B (50 min). The detection wavelength was 260 nm. The sample injection volume, oven temperature, and flow rate were set at 5 *μ*L, 30°C, and 1.0 mL·min^−1^, respectively. References standards of quercitrin, isoquercitrin, and vanillic acid were weighed and dissolved in methanol.

### 2.4. Quality Control of VOAV by Gas Chromatography-Mass Spectrometer (GC-MS)

Chromatographic analysis was performed for the quality control of VOAV using GC-MS (Agilent Technologies, Santa Clara, CA, USA) with a capillary column (HP-5MS Capillary; 30.0 mm × 0.25 mm × 0.25 *μ*m). The oven temperature was programmed as follows: an initial temperature of 80°C, which was increased to 280°C at a rate of 3°C min^−1^ and then at 20°C min^−1^ to a final temperature of 250°C and held for 20 min. Injection was conducted in split mode (20:1) at 250°C. The carrier gas helium was at a flow rate of 1.0 mL/min, and the injected sample volume was 1 *μ*L. The runtime was 35 min. The MS scan range was (m/z) 35–500 atomic mass units (AMU) under electron impact (EI) ionization (70 eV). EI source and quadrupole temperatures were set at 230°C and 150°C, respectively. The transfer line between the GC and the MS was maintained at 250°C.

### 2.5. Animals, Diets, and Groups

Eight-week-old male Sprague-Dawley rats were purchased from Chengdu, China (Certificate of Quality No. 0016254). The rats were acclimated in a controlled environment (temperature 21 ± 2°C, 60 ± 10% humidity, and a 12-h/12-h light/dark cycle) with free access to water. The whole research design was reviewed and approved by the Institutional Ethical Committee on Animal Care and Experimentation of Yunnan University Traditional Chinese Medicine (R-0620150014). All reasonable efforts were made to minimize animal suffering.

Rats were randomized into 12 groups with 10 animals per group (Table 1). These were housed 10 in a stainless steel cage containing sterile wood cuttings and sawdust as bedding in ventilated animal rooms. All rats except those in the control group were fed a high-fat diet (HFD) until the end of the experiment (16 weeks). HFD contained 1% cholesterol, 10% lard, 10% egg yolk, and 79% basic feed (moisture ≤ 10%; protein ≥ 20%; fat mix ≥ 4%; calcium: 1.0%–1.8%; phosphorus: 0.6–1.2; fiber ≤ 5%; essential amino acids ≥ 2%) (Research Diets, Suzhou, China).

These 12 groups of rats received different drug regimens. The normal control group (CON group, fed with normal diet) and MOD Group (fed with HFD) received only physiological saline. WEAV groups (WEAV.L, WEAV.M, and WEAV.H group) received 48 mg/kg, 96 mg/kg, and 192 mg/kg of WEAV orally, respectively. VOAV groups (VOAV.L, VOAV.M, and VOAV.H group) received 8 mg/kg, 16 mg/kg, and 32 mg/kg of VOAV, respectively. BA groups (BA.L, BA.M, and BA.H group) received 2 mg/kg, 4 mg/kg, and 8 mg/kg of BA, respectively. The ezetimibe (EZE group, 1 mg/kg) was used as positive control. Ezetimibe was used as a control positive drug because it was frequently used in the treatment of NAFLD. Ezetimibe could selectively inhibit the small intestine cholesterol transporter and effectively reduce intestinal cholesterol absorption; therefore it decreased cholesterol levels both in the plasma and in the liver. All rats were treated by gavage once per day for 16 consecutive weeks according to the dosages listed in Table 1.

### 2.6. Assessment of Blood Lipid, Lipoprotein, and Aminotransferase Levels

Blood samples (about 1.5–2.0 mL) were collected from the retroorbital venous plexus of rats every 2 weeks and then centrifuged at 10,000 rpm for 15 min. The serum was stored at −80°C until use. Serum levels of total cholesterol (TC), triglyceride (TG), free fatty acid (FFA), low-density lipoprotein cholesterol (LDL-C), high-density lipoprotein cholesterol (HDL-C), aspartate aminotransferase (AST), and alanine aminotransferase (ALT) were determined using the enzymatic colorimetric method. Serum assay detection kits were purchased from Nanjing Jiancheng Bioengineering Co., Ltd. (Nanjing, China).

After the rats were dissected, the liver was taken and weighed. The liver index was calculated by liver mass/body mass.

### 2.7. Assessment of LPS Levels in the Hepatic Portal Vein

At the end of the experiment, the rats were sacrificed using an intraperitoneal injection of 10% chloral hydrate (3.0 mL/100 g body weight). Hepatic portal vein blood was collected using a disposable vacuum blood collector. After being left to stand for 30 min, plasma was isolated by centrifugation at 3,000 rpm for 2 min. Lipopolysaccharide (LPS) contents in all groups were evaluated using tachypleus amoebocyte lysate kits (Chinese Horseshoe Crab Reagent Manufactory, Co., Ltd., Xiamen, China).

### 2.8. Flow Cytometric Analysis of Occludin and ZO-1 Protein Levels

At the end of the 16th week, the rats were executed upon anesthesia using 10% chloral hydrate solution. The jejunums of all rats were collected, cleaned, placed in PRMI-1640 culture medium solution, and cut into pieces. Cell debris in the homogenate was removed by passing this through a 70-*μ*m filter membrane. Cells were then diluted with staining buffer to a density of 1 × 10^6^ cells/mL after blocking with 3% FBS. Shortly thereafter, the cells were incubated with anti-occludin antibody (Proteintech, Chicago, Illinois, USA) and anti-ZO-1 antibody (Proteintech, USA) for 2 h and then incubated with second antibody (fluorescein isothiocyanate) in the dark for 1 h. Finally, the expression levels of occludin and ZO-1 protein were determined using flow cytometry (FACSCalibur, Becton, Dickinson and Company, San Diego, CA, USA).

### 2.9. Assessment of Protein and Cytokine Levels in the TLR4/NF-*κ*B Pathway in the Liver

At the end of the 16th week, the rats were anesthetized with 10% chloral hydrate and sacrificed. Liver tissue samples were then immediately collected for biochemical analysis and morphologic observation. The liver samples were weighed, washed with 0.9% saline, and cut into pieces. Liver tissue samples (1 g) were homogenized in 9 mL normal saline and then centrifuged at 4,000 rpm for 10 min at 4°C. The supernatant was then collected for further analysis. AST, ALT, TG, TC, FFA, LDL-C, and HDL-C concentrations were determined in all supernatants. TLR4, TNF-*α*, IL-10, IL-1*α*, and IL-6 concentrations were tested using ELISA assay kits (Cusabio Biotech Co., Ltd., China). Protein expression levels of NF-*κ*B, IKK, and I*κ*B were determined by western blotting. Antibodies against NF-*κ*B (1:1,000), IKK (1:1,000), I*κ*B*α* (1:1,000), anti-rabbit IgG (1:10,000), and *β*-actin (1:1000) dilution were used in this study.

### 2.10. Overall Structural Changes in Gut Microbiota

The composition of the bacterial communities in each fecal sample was assessed as previously described [5, 23]. Sequencing of the variable region V4 in 16S rDNA was used in the analysis of gut microbiota species diversity in rat fecal samples.

At the end of the experiment, rat feces of the CON, MOD, EZE, WEAV.M, VOAV.M, and BA.M groups were collected in sterilized plastic tubes and stored at −80°C until testing. All fecal samples in the same group (0.5 mg for each rat) were carefully blended, and genomic DNA was extracted from 0.5-mg portions of pooled samples using SDS. The DNA extracted from fecal samples was subjected to pyrosequencing of the V4 region of 16S rDNA. PCR amplification of the primers was performed using 515f/806r. Sequencing was conducted on an Illumina MiSeq platform [24]. The regulatory role of* A. villosum* on the intestinal microecological system was indicated by the *β* diversity and OTU analysis of the fecal samples.

## 3. Statistical Analysis

The data (mean ± SD) were evaluated using one-way ANOVA at significance levels of *P* < 0.05, < 0.01, and < 0.001. Analysis of principal coordinates (PCoA) was conducted to explore and visualize similarities among different groups. The unweighted pair-group method with arithmetic mean (UPGMA) method was used for cluster analysis to assess similarities among samples.

## 4. Results

### 4.1. Chemical Profiles of WEAV and VOAV

**Figure 1:**
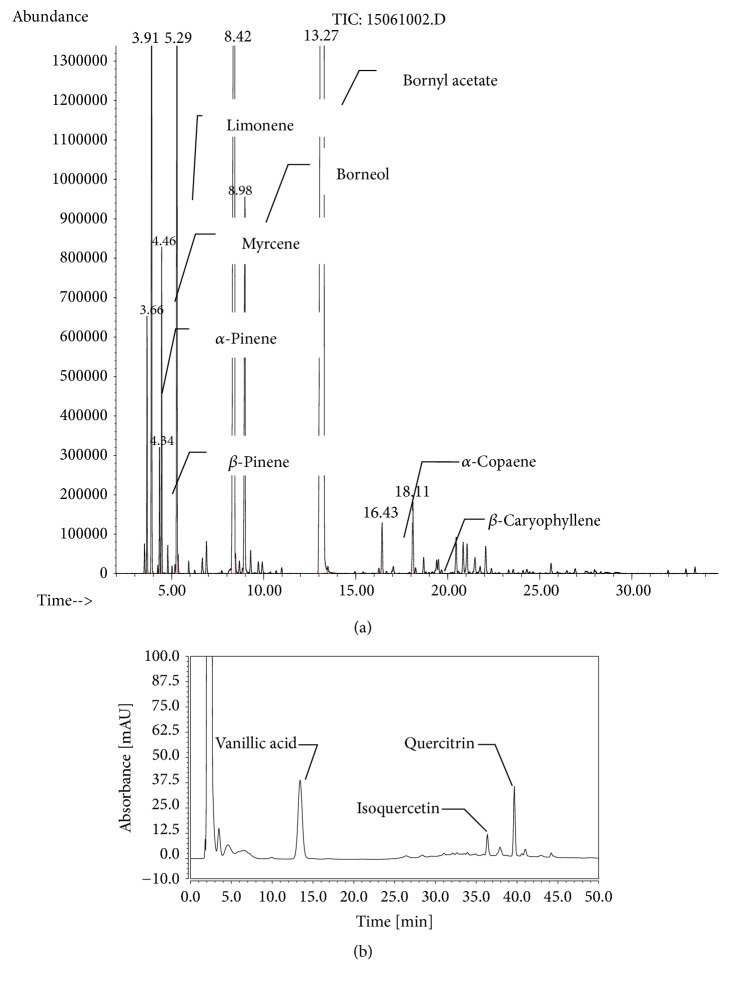
**Chemical constituents of* Amomum villosum*.** (a) GC-MS profile of the volatile oil of* A. villosum; *(b) HPLC profile of water extract of* A. villosum.*

Here, 58 volatile components were identified in VOAV by GC-MS (Figure 1(a)). The 10 most abundant components are listed in Table 2, which included bornyl acetate, camphor, camphene, limonene, borneol, myrcene, *α*-pinene, *β*-caryophyllene, *β*-pinene, and *α*-copaene. These accounted for 94.2% of the total volatile compound content in* A. villosum*. BA accounted for up to 54.54% and thus could be considered as the representative component of VOVA.

The HPLC profile of WEAV is shown in Figure 1(b). The linear ranges for quercitrin, isoquercitrin, and vanillic acid were 0.1172–0.1440 *μ*g/mL (*r* = 0.9999, *n* = 6), 0.1072–1.141 *μ*g/mL (*r* = 0.9999, *n* = 6), and 0.1040–1.120 *μ*g/mL (*r* = 0.9999, *n* = 6), respectively. Average recovery rates were 99.07% (RSD = 0.39%), 98.23% (RSD = 1.63%), and 99.31% (RSD = 0.70%), respectively. The concentrations of quercitrin, isoquercitrin, and vanillic acid in WEAV were 0.0604 mg/g, 0.0276 mg/g, and 0.1709 mg/g, respectively.

### 4.2. *A. villosum* Prevents HFD-Induced NAFLD in Rats

**Figure 2:**
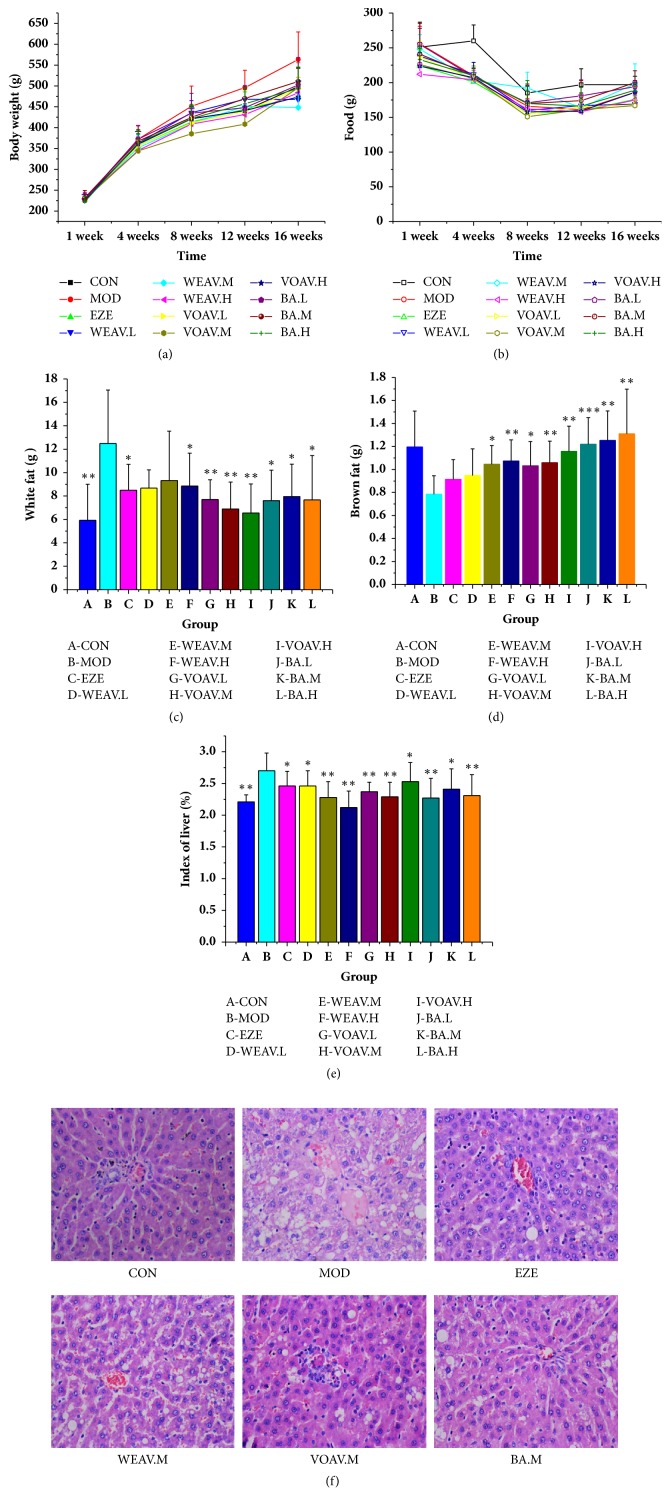
***Amomum villosum* reduced body weight and fat accumulation in HFD-fed rats.** Effects of* A. villosum* treatment on (a) body weight, (b) food intake, (c) white fat, (d) brown fat, (e) liver index, and (f) liver biopsy. (a–e) The difference between groups was evaluated using one-way analysis of variance (ANOVA, n = 10). Asterisks (^*∗*^) indicate a significant difference from the model group, ^*∗*^*P* < 0.05, ^*∗∗*^*P* < 0.01, ^*∗∗∗*^*P* < 0.001. (f) Representative images (200× magnification, hematoxylin and eosin stain) of hepatic histology in CON, MOD, EZE, WEAV.M, VOAV.M, and BA.M groups. No obvious fatty degeneration was observed in hepatocytes in the CON group. Obvious edema and steatosis were observed in hepatocytes after being fed a high-fat diet for 16 weeks. They were markedly relieved in VOVA.M and BA.M groups.

HFD feeding for 16 weeks led to a significant increase in body weight, liver index, accumulation of epididymal fat, and subcutaneous adipose tissue. HFD resulted in more enhancement in lipid deposition in adipocytes and hepatocytes than chow feeding. Simultaneously, the HFD group still had less brown fat. The treatment groups showed distinct differences from the MOD group in terms of increase in body weight, food intake, and white and brown fat weight (Figures 2(a)–2(d)). The application of WOVA, VOVA, and BA decreased the liver index raised by HFD (Figure 2(e)).

Fat degeneration, swelling of the liver cells, uneven lipid droplets, and large numbers of inflammatory cells were observed in the liver tissue of the HFD group. VOAV and BA were found to relieve fat degeneration and edema of liver cells (Figure 2(f)).

The levels of TG, TC, LDL-C, HDL-C, AST, ALT, and FFA were tested at the end of the study (Table 3). HFD feeding was associated with higher TC and TG levels in the liver than control cow feeding by 71.4% and 45.1%, respectively. These results indicated that the NAFLD model had been successfully established in the current study. The application of* A. villosum *resulted in a reduction in the levels of FFA, TG, and TC in the liver. The elevations of AST and ALT induced by HFD were also alleviated by* A. villosum *(Table 3). WEAV, VOAV, and BA effectively inhibited the FFA supply, thus maintaining hepatic TG content at normal levels. WEAV, VOAV, and BA reduced the expression of LDL-C and increased the expression of HDL-C, thereby reducing the accumulation of hepatic TC to some extent.

### 4.3. *A. villosum* Regulates the Intestinal Microbial Balance

Disturbances in the balance of intestinal flora here refer to changes in the number of intestinal flora, including strains and their relative proportions.

**Figure 3:**
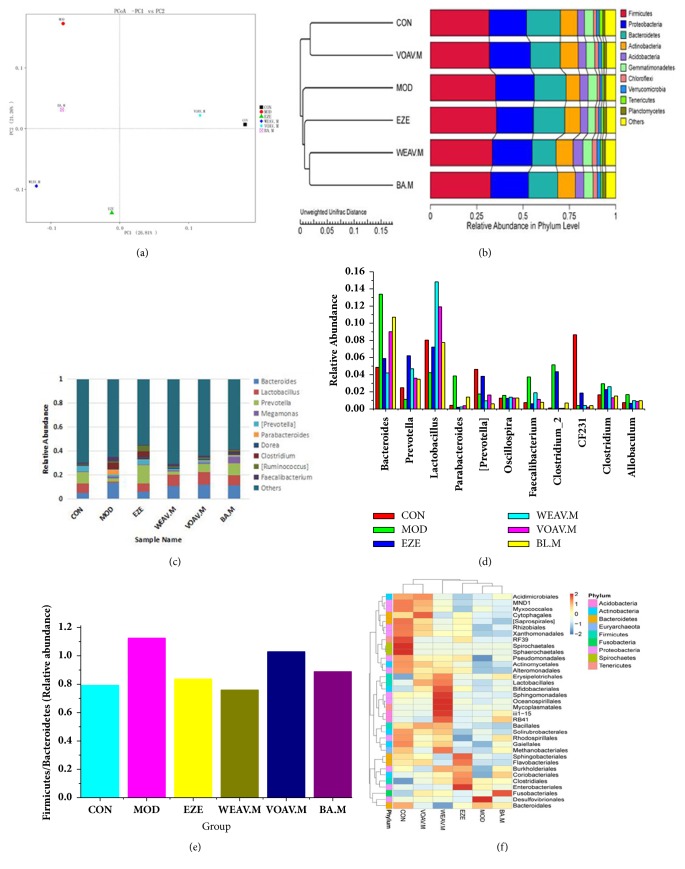
***Amomum villosum* altered the microbiota composition in HFD-fed mice. **Microbiota composition in the feces of chow-fed mice; HFD mice treated with or without* A. villosum* were analyzed using 16s rDNA pyrosequencing (n = 10 for each group). (a) UniFrac-based PCoA analysis. (b) UPGMA clustering tree. (c) Relative abundance at the genus level. (d) Relative abundances of key genera in Bacteroidetes and Firmicutes phyla. (e) Firmicutes-to-Bacteroidetes ratio. (f) Species richness on the level of dendrogram order. The horizontal axis represents the sample information, the vertical axis represents species annotation information, the left side of the cluster tree represents the species cluster tree, and the top of the cluster tree represents the sample tree.

At the end of the study, the effects of* A. villosum* on the composition of gut microbiota were assessed through a sequencing-based analysis of bacterial 16S rRNA (V4 region) in the feces. UniFrac-based PCoA revealed a distinct clustering of microbiota composition for each treatment group (Figure 3(a)). The community structure of the VOAV.M group displayed considerable similarity to the control group, thus indicating that VOAV imparts considerable regulatory effects on the intestinal flora of NAFLD rats. This result was also confirmed using the UPGMA clustering tree (Figure 3(b)).

The 10 most abundant genera were compared across groups (Figure 3(c)). The composition of intestinal bacterial species dramatically changed after the mice were fed HFD for 16 weeks. Abundances of* Lactobacillus* and* Prevotella *were reincreased in the treatment groups. The application of VOAV induced the greatest increase in the relative abundance of* Lactobacillus*. Relative abundances of key genera in the Bacteroidetes and Firmicutes phyla (Figure 3(d)) showed that* A. villosum* induced an increase in the densities of* Prevotella *and* CF231 *and reduced that of* Bacteroides* and* Parabacteroides*. These genera belong to the Bacteroidetes phylum.* A. villosum *reduced the relative abundance of some genera within the Firmicutes phylum such as* Clostridium*,* Faecalibacterium*,* Clostridium-2*,* Allobaculum*, and* Oscillospira* (Figure 3(d)). Therefore,* A. villosum* effectively ameliorates the increase in the Firmicutes-to-Bacteroidetes ratio in HFD-fed rats (Figure 3(e)).

Heatmap and cluster analysis of the 35 most abundant orders of intestinal flora were conducted based on the abundance of species annotation information (order). These treatment groups were divided into two categories: VOAV and CN were located in one cluster unit; WEAV, BA, MOD, and EZE were located in another cluster unit (Figure 3(f)). These results suggested that the microbial abundances of high-fat-intake rats dramatically differed from that of rats fed on a normal diet. VOAV restored the disturbed balance of intestinal flora to normal levels. However, BA showed different effects on intestinal flora compared with VOAV. This suggests that volatile constituents other than BA may also contribute to the activity of VOAV. After comprehensive consideration of the UniFrac-based PCoA (Figure 3(a)), the UPGMA clustering tree (Figure 3(b)), the relative abundances of genera (Figures 3(c) and 3(d)), the Firmicutes-to-Bacteroidetes ratio (Figure 3(e)), and cluster analysis of the 35 most common orders (Figure 3(f)), we concluded that VOAV has a profound regulatory effect on the HFD-induced NAFLD intestinal microecological damage.

#### 4.3.1. *A. villosum* Protects the Intestinal Mucosal Barrier and Relieves Endotoxin

**Figure 4:**
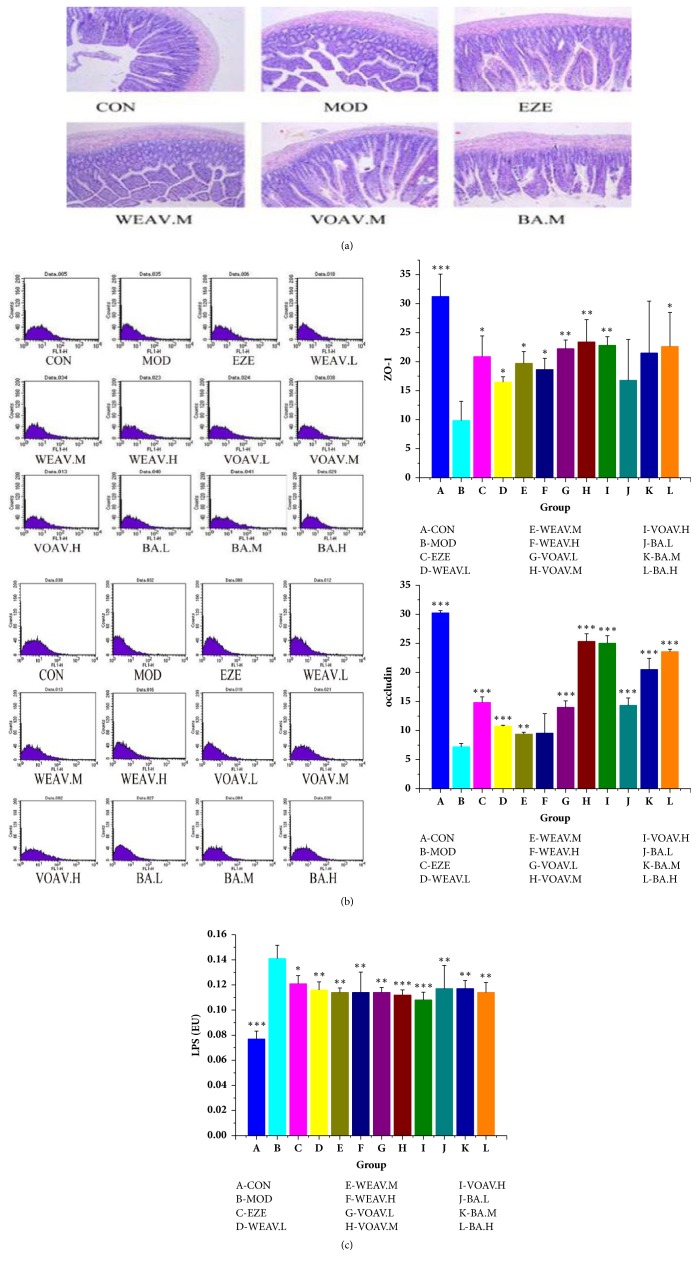
***A. villosum* protected the intestinal mucosal barrier and reduced the effects of endotoxins. **(a) Comparison of microscopic morphology in ileum tissue among CON, MOD, EZE, WEAV.M, VOAV.M, and BA.M groups. Sectional representations (100× magnification, hematoxylin and eosin stain) of ileum tissues are shown. Degeneration and necrosis of ileum were relieved after VOVA and BA treatment. (b) Concentrations of ZO-1 and occludin. (c) Concentrations of LPS in hepatic portal vein. LPS levels in hepatic portal vein blood samples were measured using a tachypleus amebocyte lysate test (mean ± SD, n = 10). Statistical significance: ^*∗*^*P* < 0.05 versus model; ^*∗∗*^*P* < 0.01 versus model; ^*∗∗∗*^*P*< 0.001 versus model.

In the present study, degeneration and necrosis in the epithelial tissue of the intestinal mucosa in NAFLD rats were observed (Figure 4(a)). Treatment of VOAV minimized the damage and maintained the structural integrity of these tissues, whereas WEAV did not.

The protein expression levels of ZO-1 and occludin were significantly lower in the model group than in the normal group (Figure 3(b)). Additionally, VOAV increased occludin protein expression to normal levels, as well as significantly increasing the protein expression of ZO-1.

The production of endotoxin by intestinal microbes can cause chronic low-grade inflammation in patients with NAFLD. After the rats were fed on an HFD for 16 weeks, significantly higher endotoxin levels were observed in the NAFLD group relative to that in the control group (Figure 4(c)).* A. villosum *inhibited the increase in LPS levels, particularly in the VOAV group (*P* < 0.001). This inhibitory effect might be related to its beneficial effects on gut microbiota equilibrium.

In general, VOAV could protect the function of intestinal mucosal barrier, increase the expression of the occludin and ZO-1 proteins, and ameliorate endotoxin translocation induced endotoxemia.

#### 4.3.2. *A. villosum* Inhibits the TLR4/NF-*κ*B Inherent Immune Response System of HFD Rats

**Figure 5:**
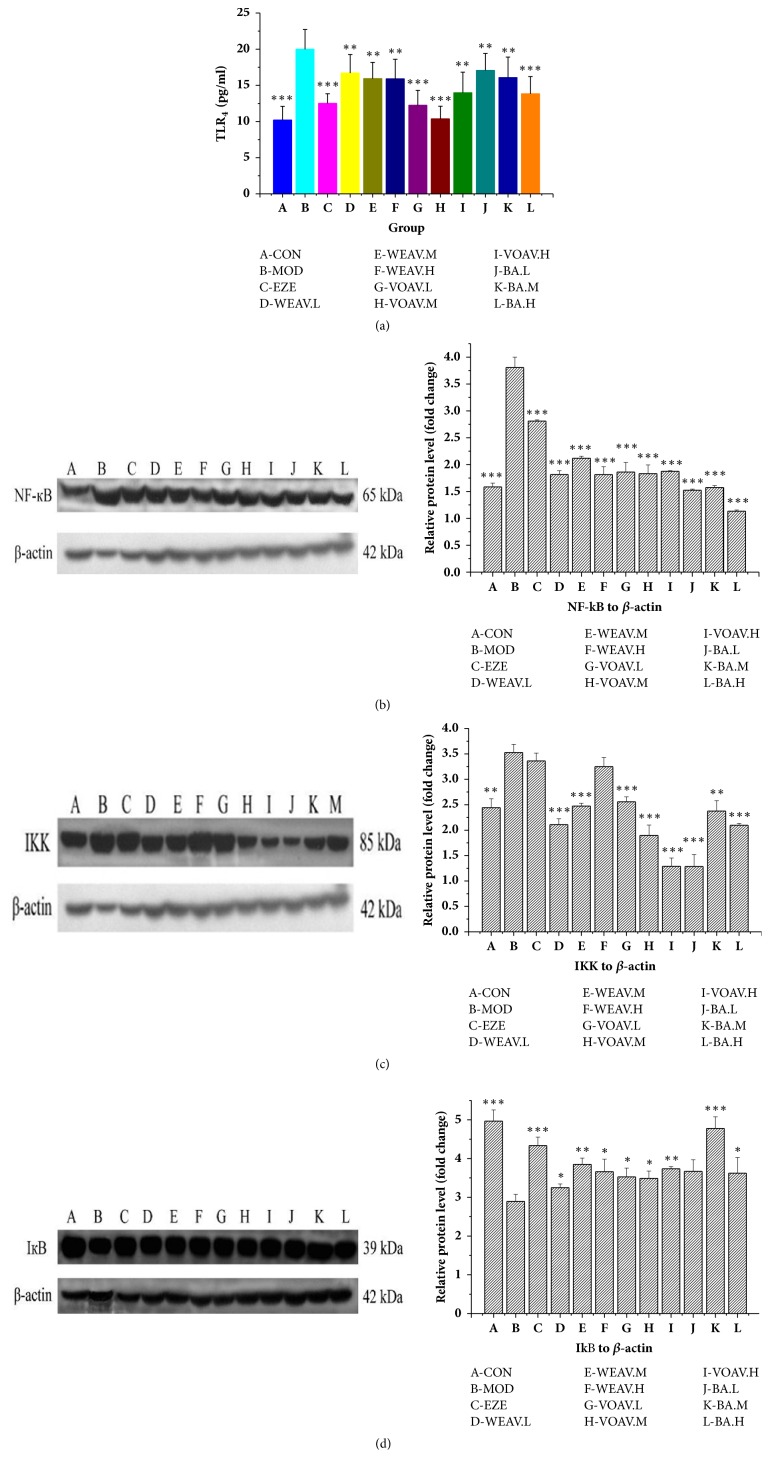
***A. villosum* reduced the TLR4/NF-**κ**B inherent immune response system in HFD rat**. Effects of* A. villosum* treatment on (a) TLR4 protein production, (b) NF-*κ*B protein production, (c) IKK protein production, and (d) IкB production were examined in liver tissue. Representative immunoblots for target proteins are shown in (b), (c), and (d). Molecular weight markers are here given in kilodaltons (kDa). Protein levels were normalized to internal controls (beta-actin). Data are shown as mean ± SD (n = 10). Statistical significance: ^*∗*^*P* < 0.05 versus model; ^*∗∗*^*P* < 0.01 versus model; ^*∗∗∗*^*P* < 0.001 versus model.

The TLR4 levels in the liver tissue of HFD-fed NAFLD rats showed a twofold increase compared to the CON group (Figure 5(a)). However, intervention using* A. villosum *effectively inhibited the expression of TLR4. The VOAV.M group showed superior performance with respect to reducing TLR4 levels in the liver, wherein expression dropped by about 50% reduced.

Figures 5(b)–5(d) show that WEAV, VOAV, and BA inhibited NF-*κ*B and IKK production in hepatic tissues of HFD-fed rats. Moreover, the production of IкB, whose interaction with NF-кB was found to prevent NF-кB translocation and activation, was also enhanced after* A. villosum* treatment.

#### 4.3.3. *A. villosum* Suppresses the Cytokine Levels Downstream of the TLR4/NF-*κ*B Pathway

**Figure 6:**
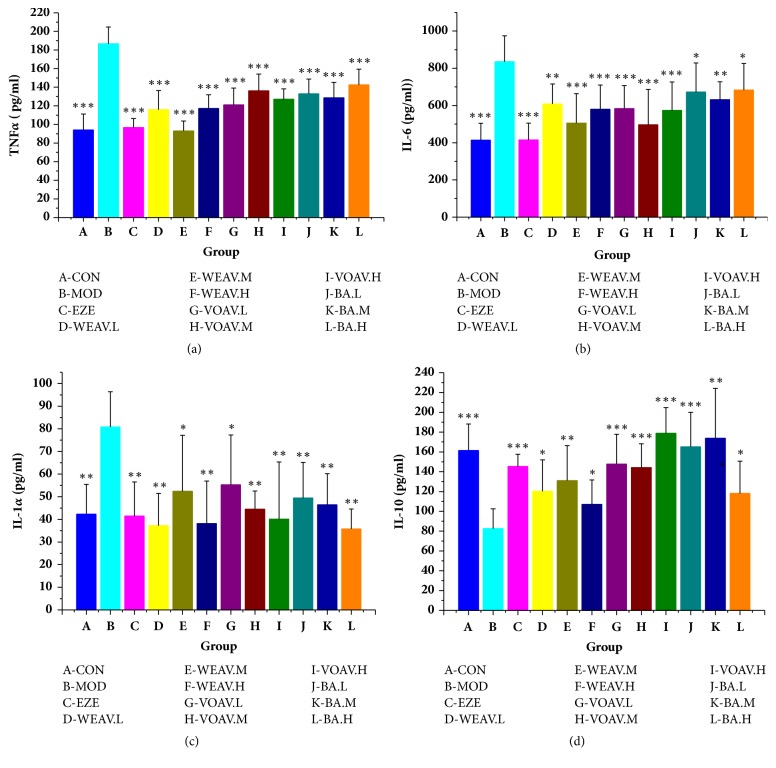
***A. villosum* suppressed the cytokine levels in the downstream of TLR4/NF-**κ**B pathway.** Relative expressions of (a) TNF-*α*, (b) IL-6, (c) IL-1*α*, and (d) IL-10 in liver tissues were assessed using ELISA and are shown relative to the MOD group. Data are shown as mean ± SD (n = 10). Statistical significance: ^*∗*^*P* < 0.05 versus model; ^*∗∗*^*P* < 0.01 versus model; ^*∗∗∗*^*P* < 0.001 versus model.

Hepatic TNF-*α*, IL-6, and IL-1*α* expression levels were higher in HFD-fed rats than in the chow-fed rats of the control group, whereas IL-10 expression was lower (Figures 6(a)–6(d)). Chronic low-grade inflammation, which is characterized by the overproduction of inflammatory cytokines such as TNF-*α*, IL-6, and IL-1*α*, was also controlled by* A. villosum* (Figures 6(a)–6(d)). In the meantime, VOAV showed a pronounced increase in hepatic IL-10 levels, which are typical anti-inflammatory cytokines. These results suggest that* A. villosum* suppresses the cytokine levels downstream of the TLR4/NF-*κ*B pathway, which was also beneficial by delaying the onset of NAFLD and limiting its development.

## 5. Discussion

Intestinal microbiota plays important roles in health and disease. Alterations in its healthy homeostasis might lead to the development of numerous liver disorders, including the complications of liver cirrhosis [25]. In recent years, numerous studies have shown that the intestinal microbiota is closely related to the development of NAFLD via the gut-liver axis. TLR4 occupies a decisive position between the signal produced in the gut and its biological effects in the liver. TLR4 is the main receptor that mediates LPS responses. LPS endotoxemia results in the upregulation of TLR4, promotes NF-*κ*B transcription, and induces the overproduction of inflammatory cytokines that induce chronic low-grade inflammation, ultimately leading to NAFLD [26].

This study was designed to explore effect of the regulatory mechanism associated with different active ingredients of* A. villosum* on lipid accumulation in NAFLD. To elucidate the mechanism by which* A. villosum *prevents lipid metabolic disorders, all rats except those in the normal group were fed HFD until the end of the experiment (16 weeks). This HFD treatment was a typical formula for high-energy intake associated with the induction of NAFLD and hyperlipidemia in rodents [27, 28].

The present study observed the following: First, In the HPLC experiment, quercitrin, isoquercitrin, and vanilla acid were used as reference products. Quercitrin and isoquercitrin were reported to have significant antioxidant activity [29]. Both quercitrin and isoquercitrin were reported to significantly reduce the levels of TG, TC, and MDA [30]. Furthermore, it was concluded that the anti-NAFLD activity of WEAV may be partly related to quercitrin and isoquercitrin.

In the meantime, a large number of literature sources reported that bornyl acetate, limonene, *α*-pinene, and *α*-copaene had obvious effects on the regulation of microbial activity [31–33], and it is speculated that regulation of intestinal microflora by VOAV was partly related to bornyl acetate, limonene, *α*-pinene, and *α*-copaene.

Second,* A. villosum* effectively inhibited the high-fat diet-induced accumulation of FFA in the liver, cutting off the supply of raw materials for endogenous TG synthesis. Simultaneously,* A. villosum* decreased TG accumulation in the liver by lowering the expression of LDL-C and increasing HDL-C content. VOAV showed the best lipid-lowering effect, which was highly similar to that in the positive control.

Third,* A. villosum* significantly regulated the balance of gut microbiota balance and the repair of intestinal mucous membrane barrier in NAFLD rats.* A. villosum* inhibited the increase in the ratio of Firmicutes and Bacteroidetes.* A. villosum* was found to reduce the relative abundance of some genera within Firmicutes such as* Clostridium*,* Faecalibacterium*,* Clostridium-2*,* Allobaculum*, and* Oscillospira*.* A. villosum* was also determined to adjust the relative abundance of some genera within the Bacteroides phylum effectively such as* Prevotella*, [*Prevotella*],* Parabacteroides*,* CF231*, and* Bacteroides *to normal levels. LPS is a major constituent of the outer membranes of gram-negative bacteria. LPS recognition and signal transmission are key events in the host defense reaction towards gram-negative bacteria and are associated with various disorders [34, 35]. VOAV was shown to prevent the translocation of endotoxin and decrease the LPS content by nearly 22%. Intestinal epithelial cells play an important role in the maintenance of intestinal epithelial cells. The integrity of the small intestinal mucosa has been observed in a variety of acute or chronic intestinal diseases [7]. Tight junction proteins of small intestinal mucosa such as zonula occludens (ZO) and occludin play critical roles in the maintenance of the intestinal epithelial barrier [36]. The protein expression levels of ZO-1 and occludin were significantly lower in the model group than in the normal group, which was consistent with previous studies [37]. The regulation of the balance of gut microbiota balance and the strong intestinal mucosal barrier mediated by* A. villosum* might be responsible for this.

Endotoxemia and TLR4 signaling control the production of proinflammatory cytokines in target tissues, which lead to chronic inflammation and insulin resistance in HFD-fed rats [38]. TLR4 is the main receptor mediating the LPS response. Nuclear factor-*κ*B (NF-*κ*B), which belongs to a family of transcription factors, is the principal player in the regulation of inflammatory gene expression. Inhibitors of NF-*κ*B (I-*κ*B) kinase (IKKs) are a central component of the signaling cascade that controls NF-*κ*B-dependent inflammatory gene transcription. IKKs activate the NF-*κ*B pathway by phosphorylating I-*κ*B and NF-*κ*B P65, making it an attractive target for therapeutic intervention [39].TLR4 and NF-*κ*B protein expression was inhibited by VOAV and WEAV.* A. villosum *was found to downregulate IKK protein expression in the TLR4/NF-кB signaling pathway. The production of IкB, whose interactions with NF-кB prevented NF-кB translocation and activation, was enhanced by* A. villosum* treatment. The release of downstream inflammatory cytokines in the LPS/TLR4/NF-*κ*B signaling pathway was found to be mitigated by* A. villosum. *Studies showed that a high-fat diet could increase NF-кB activation in mice, which led to a sustained elevation in the level of IкB kinase epsilon (IKK epsilon) in the liver, adipocytes, and adipose tissue macrophages. IKK epsilon knockout mice were protected from high-fat diet-induced obesity, chronic inflammation of the liver and fat, hepatic steatosis, and whole-body insulin resistance [40–42].

Both ezetimibe and VOAV could inhibit the increasing of TC, TG, and FFA in liver homogenates. Among them, ezetimibe displayed better inhibitory effect on the content of TC, TG, and FFA in liver homogenate (*P* < 0.001), followed by the middle-dose group of volatile oil from* Amomum villosum*. However, VOAV showed better effect on the increasing of liver AST and ALT. In the meantime,* A. villosum* could effectively inhibit lipids accumulation in circulation system.

Sequencing results of 16S rDNA suggested that both ezetimibe and VOAV inhibited the increasing ratio of Firmicutes and Bacteroidetes. Ezetimibe and VOAV could effectively regulate the intestinal microflora, relieve the chronic low-grade inflammation by promoting ZO-1 and occludin protein expressions, and inhibit TLR4/NF-кB signaling pathway. The ezetimibe was more active in regulating the accumulation of lipids in the hepatic lipids of the high-fat diet (first strike), while* A. villosum* was more effective in intervening TLR4/NF-кB upstream signaling pathway and inhibiting the expression of proinflammatory cytokines.

In sum,* A. villosum* was found to effectively inhibit lipid accumulation in liver tissue, regulate the intestinal microflora, strengthen intestinal mucous membrane barrier, inhibit the TLR4/NF-кB signaling pathway, and relieve chronic low-grade inflammation. VOAV showed great potential in NAFLD prevention and treatment. This work could provide a scientific basis for further application and development of* A. villosum. *

The present study has investigated the role of* A. villosum* in the treatment of NAFLD. Results showed that* A. villosum* could improve the permeability of intestinal epithelial cells and regulate the balance of intestinal flora. In China,* A. villosum *is used in both medicine and cuisine food [43]. This made it possible to produce a new type of intestinal microecological preparation with better lipid-lowering effects. This research also confirmed that volatile oil is more effective than* A. villosum* water extract, which could be considered scientific evidence that is in agreement with the traditional findings that* A. villosum *should be extracted promptly to prevent the oil from undergoing volatilization.

## Figures and Tables

**Table 1:** Animal grouping and treatment in this research.

Group	Diet	Treatment	Dose (mg/kg)
CON	Normal Diet	—	—
MOD	High fat Diet	—	—
EZE	High fat Diet	Ezetimibe	1
WEAV.L	High fat Diet	low dose of WEAV	48
WEAV.M	High fat Diet	middle dose of WEAV	96
WEAV.H	High fat Diet	high dose of WEAV	192
VOAV.L	High fat Diet	low dose of VOAV	8
VOAV.M	High fat Diet	middle dose of VOAV	16
VOAV.H	High fat Diet	high dose of VOAV	32
BA.L	High fat Diet	low dose of bornyl acetate	2
BA.M	High fat Diet	middle dose of bornyl acetate	4

**Table 2:** The main chemical composition and content of volatile oil.

Compound	Chemical formula	Retention time (min)	Relative content (%)
Bornyl acetate	C_12_H_12_O_2_	13.27	54.54
Camphor	C_10_H_16_O	8.42	17.92
Camphene	C_10_H_16_	3.91	6.757
Limonene	C_10_H_16_	5.30	5.249
Borneol	C_10_H_18_O	8.97	4.068
Myrcene	C_10_H_16_	4.46	1.969
*α*-Pinene	C_10_H_16_	3.67	1.503
*β*-Caryophyllene	C_15_H_24_	18.11	0.8530
*β*-Pinene	C_10_H_16_	4.34	0.7950
*α*-Copaene	C_15_H_24_	16.43	0.5430

**Table 3:** Lipid contents in the liver samples. (mean ± SD, n = 8).

	TC (mmol/L)	TG (mmol/L)	FFA (*μ*mol/L)	LDL-C (mmol/L)	HDL-C (mmol/L)	AST (U/L)	ALT (U/L)
CON	0.75 ± 0.13*∗∗∗*	1.13 ± 0.17*∗∗∗*	230.99 ± 22.83*∗∗*	1.39 ± 0.11*∗∗∗*	1.47 ± 0.16*∗∗*	2199.29 ± 114.52*∗∗∗*	1550.52 ± 157.42*∗∗∗*
MOD	1.59 ± 0.04	1.64 ± 0.11	440.22 ± 31.54	2.46 ± 0.32	1.07 ± 0.27	2856.18 ± 145.88	22242.08 ± 154.16
EZE	1.05 ± 0.14*∗∗∗*	1.31 ± 0.19*∗∗∗*	263.32 ± 21.83*∗∗*	1.49 ± 0.22*∗∗∗*	1.37 ± 0.28*∗*	2401.43 ± 99.03*∗∗*	1723.72 ± 182.66*∗∗∗*
WEAV.L	1.18 ± 0.19*∗∗*	1.45 ± 0.2	302.29 ± 33.41*∗*	1.69 ± 0.52*∗∗*	1.01 ± 0.2	2607.46 ± 58.35	2040.9 ± 145.07*∗*
WEAV.M	1.28 ± 0.20*∗∗*	1.44 ± 0.2	297.78 ± 33.45*∗∗*	1.70 ± 0.52 *∗∗*	1.06 ± 0.22	2590.23 ± 163.64*∗*	2042.8 ± 120.54*∗∗*
WEAV.H	1.15 ± 0.23*∗∗*	1.39 ± 0.14*∗∗*	276.34 ± 35.22*∗∗*	1.90 ± 0.34 *∗∗*	1.19 ± 0.35	2520.18 ± 140.79*∗*	1756.52 ± 118.37*∗∗∗*
VOAV.L	1.15 ± 0.14*∗∗*	1.46 ± 0.15*∗∗*	283.87 ± 21.89*∗∗*	1.57 ± 0.48*∗∗∗*	1.41 ± 0.35*∗*	2458.48 ± 110.67*∗∗*	1849.07 ± 131.94*∗∗∗*
VOAV.M	1.14 ± 0.21*∗∗*	1.35 ± 0.17*∗∗∗*	289.06 ± 26.28*∗∗*	1.46 ± 0.17*∗∗∗*	1.33 ± 0.31*∗*	2457.13 ± 182.37*∗∗*	1762.82 ± 116.12*∗∗∗*
VOAV.H	1.15 ± 0.18*∗∗*	1.421 ± 0.14*∗∗∗*	222.63 ± 22.24*∗∗*	1.38 ± 0.21*∗∗∗*	1.39 ± 0.27*∗*	2255.16 ± 78.83*∗∗∗*	1868.12 ± 200.21*∗∗∗*
BA.L	1.25 ± 0.18*∗∗*	1.37 ± 0.2*∗∗*	277.99 ± 22.44*∗∗*	1.83 ± 0.41*∗∗*	1.48 ± 0.42*∗*	2616.1 ± 153.52*∗*	1858.3 ± 131.9*∗∗∗*
BA.M	1.15 ± 0.23*∗∗*	1.46 ± 0.09*∗∗*	270.67 ± 26.81*∗∗*	1.69 ± 0.51*∗∗*	1.31 ± 0.36	2455.19 ± 91.44*∗∗*	1848.72 ± 156.98*∗∗∗*
BA.H	1.17 ± 0.14*∗∗*	1.32 ± 0.14*∗∗∗*	268.17 ± 33.29*∗∗*	1.67 ± 0.34*∗∗∗*	1.32 ± 0.17*∗*	2331.97 ± 167.89*∗∗∗*	1803.57 ± 129.19*∗∗∗*

*∗*indicates a significant difference compared with model group; *∗*P < 0.05, *∗∗*P < 0.01, *∗∗∗*P < 0.001.

## Data Availability

All data are contained and described within the manuscript. The datasets used and/or analyzed during the current study are available from the corresponding author on reasonable request.

## Abbreviations

ALT: Alanine aminotransferase
AST: Aspartate aminotransferase
BA: Bornyl acetate
FFA: Free fatty acid
GC-MS: Gas chromatography-mass spectrometer
HDL-C: High-density lipoprotein cholesterol
HPLC: High-performance liquid chromatography
HFD: High-fat diet
IL-10: Interleukin-10
IL-1*α*: Interleukin-1 alpha
IL-6: Interleukin-6
IKK: Inhibitor of nuclear factor-kappa-B kinase
IкB: Inhibitors kappa-B
LDL-C: Low-density lipoprotein cholesterol
LPL: Lipoprotein lipase
LPS: Lipopolysaccharide
NAFLD: Nonalcoholic fatty liver disease
NF-кB: Nuclear factor-kappa-B
OTUs: Operational taxonomic units
PBS: Phosphate buffer saline
PCoA: Principal coordinates analysis
SIBO: Small intestinal bacterial overgrowth
TC: Total cholesterol
TG: Triglyceride
TLR4: Toll-like receptor 4
TNF-*α*: Tumor necrosis factor alpha
UPGMA: Unweighted pair-group method with arithmetic mean
VLDL: Very-low-density lipoprotein
VOAV: Volatile oil of* A. villosum*
WEAV: Water extract of* A. villosum*
ZO-1: Zonula occludens-1.
